# Quality of Life and Symptom Burden in Non-Small-Cell Lung Cancer Patients Receiving Second-Line Chemotherapy Compared with Immunotherapy

**DOI:** 10.3390/medicina60111845

**Published:** 2024-11-09

**Authors:** Christos Stylianou, Ioannis Kalemikerakis, Theocharis Konstantinidis, Alkmena Kafazi, Nektarios Alevizopoulos, Stelios Parissopoulos, Ourania Govina

**Affiliations:** 1 Department of Nursing, University of West Attica, 12243 Athens, Greece; cstylianou@uniwa.gr (C.S.); ikalemik@uniwa.gr (I.K.); spariss@uniwa.gr (S.P.); 2 Department of Nursing, Hellenic Mediterranean University, 71410 Heraklion, Greece; harriskon@hmu.gr; 3 Department of Nursing, National and Kapodistrian University of Athens, 11527 Athens, Greece; meniakfz@nurs.uoa.gr; 4 Department of Oncology, Evaggelismos Hospital, 10676 Athens, Greece; nalevizopoulos@gmail.com

**Keywords:** chemotherapy, immunotherapy, non-small-cell lung cancer, symptoms, quality of life

## Abstract

*Background and Objectives*: The burdened symptomatology accompanying advanced non-small-cell lung cancer (NSCLC) is associated with poor prognosis and lower quality of life (QoL). Although both chemotherapy and immunotherapy increase survival, they are still associated with reduced functionality due to their toxicity. This study aimed to estimate the QoL and symptom burden of NSCLC patients receiving second-line chemotherapy compared to patients receiving second-line immunotherapy. *Materials and Methods*: This comparative, prospective study, conducted from January 2020 to December 2021, included 111 NSCLC patients who were divided into two groups: 61 patients receiving second-line chemotherapy and 50 patients receiving second-line immunotherapy. Patients’ QoL and symptom burden were estimated using the European Organisation for Research and Treatment of Cancer Quality of Life Questionnaire Core 30 (EORTC QLQ C-30) (value range 0–100) from treatment cycle 1 to 6. *Results*: The QoL (mean score > 50) and functionality dimensions (mean score > 50) were moderate to good in both treatment groups, while the symptom burden did not appear to be a serious problem (mean score < 50). From cycle 3 to cycle 5, QoL was significantly better in the immunotherapy group. From cycle 3, the role and social functioning scores were higher in the immunotherapy group, while emotional and cognitive functioning were higher from cycle 2 (*p* <0.05). The chemotherapy group experienced higher levels of nausea/vomiting, constipation and financial difficulties in all the cycles (*p* < 0.05). Fatigue and appetite loss were significantly greater from cycle 2 and insomnia was significantly greater from cycle 3. On the contrary, the immunotherapy group experienced higher levels of diarrhea in cycles 5 and 6 (*p* < 0.05). *Conclusions*: Although both therapy groups did not report significantly impaired QoL and severe symptoms, it seems that QoL improved in the immunotherapy group, which reported a lower symptom burden compared to the chemotherapy group.

## 1. Introduction

According to the International Agency for Research on Cancer, there were almost 20 million new cases of cancer and 9.7 million deaths from cancer in the year 2022. Lung cancer was the most frequently diagnosed cancer, responsible for one in eight cancers (12.4%) worldwide. Also, lung cancer was the leading cause of cancer death, with an estimated 1.8 million deaths (18.7% of all cases). Among men, lung cancer is the most commonly diagnosed cancer in 33 countries and the leading cause of cancer death in 89 countries [1]. Among women, lung cancer is the leading cause of cancer death in 23 countries. During the first year, the survival of patients with lung cancer, regardless of stage, amounts to approximately 41%. This percentage drops below 13% in five years [2]. 

Non-small-cell lung cancer (NSCLC), often diagnosed as advanced or metastatic disease, represents approximately 84% of all lung cancers, whereas small-cell lung cancer represents approximately 13% [3]. Historically, NSCLC has had a poor prognosis, particularly in the later stages, remaining one of the leading causes of cancer-related deaths among men and women [3]. Progression-free survival often remains unsatisfactory and, furthermore, patients are generally symptomatic and clinically vulnerable [4,5,6,7]. Patients can experience symptoms specific to their disease process (such as cough or dyspnea) or more generalized symptoms (such as fatigue and loss of appetite) [4,5,6,7]. However, treatment has changed markedly in the past decade with wider lung cancer screening, improved radiation techniques and treatment advances. These changes have likely resulted in the reported decline in NSCLC mortality [3,4,5,6,7].

Given the incurable nature of metastatic NSCLC, the goals of oncology healthcare professionals should not only focus on controlling the disease, but should also be directed at optimizing the patient’s quality of life (QoL). Health-related QoL (HRQoL) is a multi-dimensional concept that addresses the functional effect of a health status and/or the patient’s treatment. It refers to physical, role, emotional, social, cognitive, sexual and spiritual functioning on individual levels [8,9,10]. HRQoL data are a core element of treatment comparisons, support daily clinical treatment decision-making, improve communication between patients and clinicians and facilitate clinical and economic evaluations to define the most efficient allocation of healthcare resources [11]. Patient-reported HRQoL data aid clinicians to understand better toxicity and symptoms experienced by patients, as subjective symptoms, such as fatigue and pain, are frequently under-reported [12].

Chemotherapy seems to improve QoL when compared to best supportive care, likely due to better overall physical functioning and alleviation of disease-related symptoms [13]. However, the administration of subsequent lines of chemotherapy after the progression of the disease has been associated with worse outcomes with regards to physical conditioning and symptom burden [14]. Despite efforts to maximize QoL, patients unfortunately suffer a significant amount of therapy-related adverse effects as a consequence of their treatment regimen. In recent years, immunotherapy has proven to be an effective treatment for patients with NSCLC, outperforming chemotherapy in terms of response rate, overall survival, progression-free survival and safety [15,16,17,18,19,20,21,22,23].

Although it is commonly assumed that a lower frequency of adverse events equates to a better overall QoL, few studies have examined its change over time in NSCLC patients, depending on treatment type. Patients with advanced or metastatic NSCLC will likely be on treatment for the rest of their lives, and therefore the impact of disease-related symptoms and treatment-related side effects should be accounted for when evaluating HRQoL. Understanding the patient experience of disease symptoms and treatment-related adverse events is important in improving clinical outcomes and HRQoL [15,16,17,18,19,20,21,22,23].

Τhis comparative, prospective, non-randomized follow-up study aimed to estimate the QoL and symptom burden of NSCLC patients receiving second-line chemotherapy compared to patients receiving second-line immunotherapy.

## 2. Materials and Methods

### 2.1. Patient Population

The sample consisted of all patients with NSCLC undergoing second-line chemotherapy or immunotherapy in one-day clinics of two hospitals in Athens between January 2020 and December 2021. The inclusion criteria were patients of both genders, male or female, subjects over 18, with a documented NSCLC diagnosis, receiving only second-line chemotherapy or second-line immunotherapy, being able to communicate in Greek and having adequate cognitive function.

The exclusion criteria included receiving chemotherapy and immunotherapy in combination, as well as having a recognized mental health issue. 

Out of 125 patients who met the inclusion criteria, 14 refused to participate (response rate 88.8%), mainly due to lack of time. Finally, 111 patients participated: 61 patients in the second-line chemotherapy group and 50 in the second-line immunotherapy group. The first group received docetaxel or pemetrexed, while the second group received nivolumab or pembrolizumab. The demographic characteristics and clinical data related to the disease and treatment were obtained from the patients and their medical records.

The study was conducted according to the guidelines of the Declaration of Helsinki and approved by the two hospitals’ Ethics Committees. Written informed consent was obtained from all the patients before their participation in this study.

### 2.2. Functionality Assessment

The Eastern Cooperative Oncology Group (ECOG) scale was applied to assess functionality, classifying patients according to functional impairment. Scores from 0 to 4 rank five categories of physical function and ability. A score of 0 represents the condition of a patient who is able to perform all the activities without limitations, while a score of 4 represents a patient who is completely unable to do anything or take care of him- or herself. As the degree of the scale increases, so does the disorder of the patient’s functionality [24].

### 2.3. Quality of Life and Symptom Burden Assessment

To assess the QoL and symptom burden, the European Organisation for Research and Treatment of Cancer Quality of Life Questionnaire Core 30 (EORTC QLQ C-30), version 3.0 [25], was used after obtaining the relevant permission. The questionnaire was validated in the Greek language [26] and completed by the patients. It consists of 30 questions and includes functioning (physical, role, emotional, cognitive and social), symptoms (fatigue, nausea/vomiting, pain, dyspnea, insomnia, appetite loss, constipation, diarrhea and financial difficulties) and global health/total QoL scales. The results of each scale are presented with a range of values from 0 to 100. Higher functioning and global health/total QoL scale values represent a higher level of functioning and QoL, respectively, while high ratings on the symptom scale represent greater distress from the symptoms/problems.

The patients included in the study were assessed in six different time periods, before the first cycle of second-line therapy, and before each of the six following cycles. 

### 2.4. Statistical Analysis

Means and standard deviations (SDs) were used to describe the quantitative variables. Absolute (N) and relative (%) frequencies were used to describe the qualitative variables. To compare the proportions, Pearson’s χ^2^ test or Fisher’s exact test was used, where necessary. Student’s *t*-test or the non-parametric Mann–Whitney test were used to compare the quantitative variables between the two groups. Significance levels were two-sided, and statistical significance was set at 0.05. The statistical programs SPSS 26.0 and STATA 11.0 were used for the analysis.

## 3. Results

Table 1 provides the baseline demographic and clinical variables of the patients with NSCLC. This study included 111 patients, 61 of them (17 females and 44 males) receiving second-line chemotherapy and 50 of them (10 females and 40 males) receiving second-line immunotherapy, with a mean age of 67 ± 10.1 and 66 ± 8.2, respectively. There was no statistically significant difference between the groups regarding their age, gender, marital status, education level and income status. Also, the two groups did not differ in terms of clinical characteristics, such as years since NSCLC diagnosis and disease stage. In the total sample, all the study patients received platinum-based chemotherapy as a first-line therapy. Also, the majority of patients had received radiotherapy (74.8%), while 42.3% had undergone surgical resection, with no statistically significant difference between the two groups. However, there were significantly more patients with metastases in the chemotherapy group, compared to the immunotherapy group (*p* = 0.050) (Table 1). 

For all the samples, the mean value of the ECOG scale was 0.69 ± 0.89 in the baseline assessment. The functional assessment of the participants by type of second-line therapy is presented in Table 2. The score on the ECOG scale was similar between the two groups of patients in all the treatment cycles.

According to the EORTC QLQ-C30 scale, the mean value of the global health status/total QoL was above 50 points for all the patients in all the treatment cycles. The data regarding global health/total QoL differences between the two groups are presented in Table 3. In cycles 1 and 2, there were no significant differences in the QoL score between the patients receiving chemotherapy and those receiving immunotherapy. The QoL was significantly better in the immunotherapy patients from treatment cycle 3 (mean difference 18 points) to treatment cycle 5 (mean difference 18.8 points), while in cycle 6 it was similar in both groups.

According to the EORTC QLQ-C30 scale, all the functioning dimensions were above 50 points for all the patients in all the treatment cycles. The highest mean score was recorded in cognitive functioning (92.2 ± 15.1), followed by emotional (85.5 ± 17.2), physical (72.6 ± 27.5), social (69.7 ± 32.6) and role (66.1 ± 34.3) functioning. Table 4 presents data regarding the functional scale score differences between the two groups. In cycle 1,there were no significant differences in the functional scales scores between the patients receiving chemotherapy and the patients receiving immunotherapy. Physical functioning was similar in the two groups of patients throughout the treatment cycles. The role functioning score, as well as the social functioning score, was significantly higher in patients receiving immunotherapy from cycle 3 onwards, while the emotional and cognitive functioning scores were significantly higher in the patients receiving immunotherapy from cycle 2 onwards.

Throughout the study, patients reported more symptoms of insomnia (mean score 39.3 ± 33.0), appetite loss (mean score 32.2 ± 32.5) and fatigue (mean score 27.3 ± 26.5) and a lower burden of nausea/vomiting (mean score 7.6 ± 11.9) and diarrhea (mean score 7.8 ± 18.2). The mean score for all the symptom scales was below 50 points for all the patients in all the treatment cycles. The data regarding symptom scale score differences between treatment groups are presented in Table 5. Symptoms of fatigue and loss of appetite were significantly greater in the patients receiving chemotherapy from cycle 2 onwards. Symptoms of nausea/vomiting and constipation, as well as financial difficulties, were significantly more in the patients receiving chemotherapy in all the treatment cycles. However, pain and dyspnea were similar in the two groups in all the cycles. Insomnia symptoms were significantly greater in the patients receiving chemotherapy from cycle 3 onwards, while diarrhea symptoms were significantly greater in the patients receiving immunotherapy during cycles 5 and 6.

## 4. Discussion

Despite the underreporting of QoL in clinical studies [27,28], its measurement using self-completed instruments can contribute to improving patient care by highlighting needs, such as emotional and spiritual well-being, that would otherwise be overlooked [29]. The main results of our study show that patients with NSCLC reported good overall QoL and cognitive, emotional and physical functioning. The scores we recorded are considered higher compared to other studies of cancer patients [30,31,32,33]. In a Greek study that examined the quality of life of 95 cancer patients, the general health/QoL was moderate (62.6) and, similarly to our study, the highest scores were recorded in cognitive (75.4), physical (66.8) and emotional functioning (66.6) [28]. According to another Greek study with a sample of lung cancer patients, the QoL of 168 patients with NSCLC was even lower (57.3), while these patients also reported better emotional (68.6), cognitive (61.8) and physical functioning (57.2), compared to role (49.2) and social functioning (49.9) [32]. These differences can be partially explained by differences in sample size and data collection location, as well as by the patients’ different demographic and clinical characteristics.

Regarding QoL, depending on the type of treatment, our findings show that in contrast to the group receiving chemotherapy, the patients receiving immunotherapy reported significantly better general health status from the 3rd to the 5th cycle of treatment, with the difference between the two groups being greater in the 5th cycle. Furthermore, from cycle 3 onwards, it appears that while the immunotherapy group responded better to the demands of the disease, as well as their participation in social functioning, the chemotherapy group reported a reduced role and social functioning. These differences suggest that immunotherapy is probably a better option for patients with advanced NSCLC, as it not only does not worsen but improves functional scales. In addition, higher emotional and cognitive functioning in the immunotherapy group from cycle 2 onwards highlights the differential effects by treatment group. Our findings agree with previous international studies, which report a better QoL based on the EORTC QLQ-C30 scale in patients with NSCLC receiving second-line immunotherapy, compared to patients receiving second-line chemotherapy [34,35,36]. The difference in the mean change in EORTC QLQ score was in favor of the immunotherapy group from 2.7 to 7.8 points (*p* < 0.05) [34,35,36]. These differences might have been greater if the data analysis had included patients who had discontinued treatment, who usually have a lower QoL [37].

Although chemotherapy remains a key component in the treatment of NSCLC, it is associated with a lower response rate and overall survival, as well as reduced functionality, mainly due to the symptoms it causes [38,39,40,41]. Unlike traditional chemotherapy, immunotherapy significantly improves long-term survival but does not rule out the occurrence of side effects. Therefore, the assessment of HRQoL is of utmost importance in NSCLC patients to balance the benefits and potential risks associated with treatment. Patients’ expectations about treatment and its impact in QoL play an increasing role in treatment selection and drug evaluation [38,39,40,41].

Regarding the symptoms related to the disease and its treatment, in all the phases of our study, the patients reported a mean score of <50 on the EORTC QLQ-C30 symptom scales. The highest mean score was observed for insomnia, loss of appetite and fatigue. Conversely, the lowest mean score was observed for nausea/vomiting and diarrhea. Our results are considered to be similar to other studies in patients with cancer or NSCLC [31,32,42,43]. More specifically, 334 NSCLC patients reported a similar mean symptom score (27.2), while the predominant symptoms were dyspnea, fatigue and insomnia [42]. On the other hand, regarding 230 NSCLC patients, the lowest scores were observed for diarrhea and nausea/vomiting [43].

From the comparative analysis of symptoms by treatment group, it appears that immunotherapy was better tolerated, as the symptoms of fatigue and insomnia remained stable, while they increased significantly in the patients receiving chemotherapy. In addition, the constipation score was reduced in the patients receiving immunotherapy, with no change in the chemotherapy group. Contrary to previous findings, diarrheal symptoms increased significantly in the immunotherapy group, while they did not change in the chemotherapy group. The differences in favor of immunotherapy might have been greater if symptom assessment had not been carried out before starting treatment, as several docetaxel-related symptoms were expected to worsen after drug administration [44].

Similar multicenter studies show that there was a statistically significant improvement in the immunotherapy group compared to the chemotherapy group, confirming the better tolerance of immunotherapy compared to chemotherapy [16,35,36,45]. The greatest improvement was observed in alopecia (difference between groups −50.5 to −18.5 points), followed by fatigue (difference between groups −15.9 to −4.5 points), peripheral neuropathy (difference between groups −12.9 to −8.4 points), loss of appetite (difference between groups −11.9 to −7.5 points), dyspnea (difference between groups −11.7 to −7.0 points) and cough (difference between groups −7 to −4 points) [16,35,36,45].

Many studies have reported that physical symptoms, such as dyspnea, fatigue, pain and insomnia, negatively affect the QoL of NSCLC patients, while social support and resilience are associated with improved QoL [4,5,6,7,46]. Therefore, oncology healthcare professionals should include in patients’ care plan not only symptom relief, but resilience and social support.

A limitation of this study was the small sample size, which is often observed in non-multicenter studies. Additionally, due to the non-random sampling, it is difficult to generalize the study’s findings, as non-randomization does not rule out the possibility that factors other than treatment may be related to the QoL and symptom burden.

## 5. Conclusions

In conclusion, our study findings demonstrated a good general health status/QoL in NSCLC patients, and despite the varied symptoms related to the disease and its treatment, they reported a moderate burden, with the predominant symptoms being insomnia, loss of appetite and fatigue. Comparative analysis of the data showed that QoL improved in the immunotherapy group, who reported a lower burden from most symptoms compared to the chemotherapy group.

The present study highlights the need for multicenter studies that will evaluate QoL in larger populations of patients with NSCLC, emphasizing that patients’ reports on QoL should be considered necessary to develop new health strategies that could improve the level of health care provided, in terms of the treatment, rehabilitation or palliative care of oncology patients.

## Figures and Tables

**Table 1:** Sample characteristics and differences between patients receiving second-line chemotherapy and immunotherapy.

Data	All Patients (N = 111)	Chemotherapy Group (N = 61)	Immunotherapy Group (N = 50)	*p* +
Gender, males	84 (75.7%)	44 (72.1%)	40 (80%)	0.336
Age, years (mean, SD)	66.5 (9.2)	67 (10.1)	66 (8.2)	0.593
Marital status				0.059
Single	6 (5.4%)	6 (9.8%)	0 (0%)	
Married	70 (63.1%)	36 (59%)	34 (68%)	
Divorced	11 (9.9%)	4 (6.6%)	7 (14%)	
Widow/widower	24 (21.6%)	15 (24.6%)	9 (18%)	
Educational level				0.580
Primary school	57 (51.4%)	28 (45.9%)	29 (58%)	
High school	28 (25.2%)	17 (27.9%)	11 (22%)	
University	26 (23.4%)	16 (26.2%)	10 (20%)	
Income status				0.199
Bad/poor	33 (29.7%)	22 (36.1%)	11 (22%)	
Adequate	49 (44.1%)	24 (39.3%)	25 (50%)	
Good	29 (26.1%)	15 (24.6%)	14 (28%)	
Years since diagnosis				0.211
2	25 (22.5%)	11 (18%)	14 (28%)	
3	86 (77.5%)	50 (82%)	36 (72%)	
Disease stage				0.499
2	1 (0.9%)	1 (1.6%)	0 (0%)	
3	48 (43.2%)	24 (39.3%)	24 (48%)	
4	62 (55.9%)	36 (59%)	26 (52%)	
Metastases	85 (76.6%)	51 (83.6%)	34 (68%)	0.050
Platinum-based first-line chemotherapy	111 (100%)	61 (100%)	50 (100%)	N/A
Previous radiotherapy	83 (74.8%)	42 (68.9%)	41 (82%)	0.113
Previous surgical resection	47 (42.3%)	27 (44.3%)	20 (40%)	0.651

+ chemotherapy vs. immunotherapy; SD = Standard Deviation; N/A = Not Applicable.

**Table 2:** Functionality differences between therapy groups.

	ECOG Scale Score (Mean, SD)		
Treatment Cycle	Chemotherapy Group (N = 61)	Immunotherapy Group (N = 50)	*p*
1	0.7 (0.94)	0.68 (0.84)	0.934
2	0.89 (1.16)	0.84 (1.06)	0.959
3	1.08 (1.33)	0.9 (1.11)	0.600
4	1 (1.21)	0.73 (0.99)	0.409
5	0.86 (1.14)	0.76 (0.94)	0.882
6	0.63 (0.93)	0.62 (0.92)	0.994

SD = standard deviation.

**Table 3:** Global health/total quality of life differences between therapy groups.

	Global Health/Total QoL(Mean, SD) Range 0–100			
Treatment Cycle	Chemotherapy Group (N = 61)	Immunotherapy Group (N = 50)	Between-Groups Difference	*p*
1	68.6 (28.4)	77.7 (31.2)	−9.1	0.111
2	63.4 (31.3)	74.8 (34.7)	−11.4	0.071
3	56.8 (33.8)	74.8 (35.7)	−18.0	0.008
4	63.0 (28.9)	79.8 (29.6)	−16.8	0.007
5	63.3 (32.4)	82.1 (27.0)	−18.8	0.007
6	75.6 (25)	86.5 (25.4)	−10.9	0.094

QoL = quality of life; SD = standard deviation.

**Table 4:** Functional scales score differences between therapy groups.

		Treatment Cycles					
Functional Scales (Mean, SD) Range 0–100	Treatment Groups	1	2	3	4	5	6
Physical functioning	CMT (N = 61)	75.5 (24.9)	68.0 (29.4)	62.4 (33.4)	65.6 (27.4)	68.4 (29.6)	72.6 (25.8)
	IMT (N = 50)	77.1 (25.1)	71.6 (29.7)	72.4 (30.5)	75.9 (28.3)	79.8 (23.1)	82.2 (22.9)
	*p*	0.746	0.522	0.105	0.085	0.062	0.123
Role functioning	CMT (N = 61)	66.1 (35.7)	58.2 (36.6)	52.2 (37.9)	57.8 (36.7)	60.0 (37.1)	63.0 (34.1)
	IMT (N = 50)	71.4 (32.6)	68.0 (36.2)	67.3 (37)	73.3 (32.1)	76.8 (28.6)	79.7 (28.1)
	*p*	0.423	0.161	0.037	0.035	0.029	0.035
Emotional functioning	CMT (N = 61)	81.7 (20.0)	79.9 (20.8)	77.8 (22.0)	80.8 (18.7)	81.7 (20.5)	82.8 (21.5)
	IMT (N = 50)	87.7 (16.9)	89.2 (15.5)	88.2 (17.3)	90.0 (15.6)	92.9 (9.2)	93.5 (9.0)
	*p*	0.098	0.011	0.007	0.013	0.002	0.008
Cognitive functioning	CMT (N = 61)	90.7 (16.3)	87.4 (21.4)	81.2 (27.0)	86.7 (21.8)	86.2 (25.7)	94.4 (13.9)
	IMT (N = 50)	95.3 (11.2)	96.0 (10.9)	96.0 (10.9)	98.2 (7.3)	97.2 (8.3)	98.2 (7.6)
	*p*	0.091	0.012	<0.001	0.001	0.012	0.171
Social functioning	CMT (N = 61)	67.8 (31.8)	62.3 (36.8)	53.6 (37.8)	57.8 (34.9)	62.4 (36.9)	65.4 (31.7)
	IMT (N = 50)	77.3 (28.1)	72.7 (34.8)	73.3 (34.3)	79.3 (30.4)	82.1 (25.9)	82.9 (27.9)
	*p*	0.099	0.133	0.005	0.003	0.008	0.023

SD = standard deviation; CMT = chemotherapy; IMT = immunotherapy.

**Table 5:** Symptom scales score differences between therapy groups.

		Treatment Cycles					
Symptom Scales (Mean, SD) Range 0–100	Treatment Groups	1	2	3	4	5	6
Fatigue	CMT (N = 61)	25.1 (26.1)	35.2 (29.6)	41.2 (29.1)	37.8 (24.9)	37.1 (25.3)	35.0 (25.7)
	IMT (N = 50)	20.9 (28.5)	22.2 (29.9)	23.8 (29.3)	18.8 (25.7)	17.9 (23.8)	13.5 (21.1)
	*p*	0.108	0.005	0.001	<0.001	<0.001	<0.001
Nausea and vomiting	CMT (N = 61)	9.6 (14.4)	11.5 (13.8)	14.5 (16.5)	13.0 (16.2)	12.9 (16.7)	11.7 (15.2)
	IMT (N = 50)	2.7 (7.0)	3.0 (8.0)	5.3 (12.3)	3.7 (10.6)	2.4 (7.0)	1.4 (6.1)
	*p*	0.004	<0.001	<0.001	<0.001	0.001	<0.001
Pain	CMT (N = 61)	19.7 (27.5)	24.0 (30.7)	30.3 (31.8)	27.4 (30.4)	26.2 (35.5)	22.2 (31.0)
	IMT (N = 50)	22.7 (33.3)	23.7 (33.0)	23.7 (34.0)	18.5 (30.4)	18.3 (27.8)	20.3 (28.6)
	*p*	0.867	0.810	0.135	0.089	0.387	0.822
Dyspnea	CMT (N = 61)	23.5 (31.2)	30.1 (33.7)	37.2 (34.5)	29.6 (30.3)	25.7 (28.1)	21.0 (32.2)
	IMT (N = 50)	22.0 (29.8)	21.3 (28.4)	25.3 (31.3)	24.4 (30.5)	17.9 (25.9)	13.5 (22.9)
	*p*	0.775	0.176	0.065	0.373	0.195	0.432
Insomnia	CMT (N = 61)	39.9 (33.2)	45.4 (34.4)	54.1 (30.5)	50.4 (32.3)	45.7 (30.3)	45.7 (28.0)
	IMT (N = 50)	29.3 (31.3)	34.0 (37.2)	36.0 (37.4)	34.1 (35.2)	29.3 (33.5)	27.9 (32.9)
	*p*	0.100	0.092	0.010	0.029	0.027	0.021
Appetite loss	CMT (N = 61)	30.1 (32.6)	44.3 (30.9)	45.4 (31.6)	43.7 (30.0)	47.6 (34.6)	50.6 (33.8)
	IMT (N = 50)	22.7 (31.9)	26 (37.7)	27.3 (38.5)	18.5 (30.6)	15.5 (28.0)	15.3 (30.0)
	*p*	0.180	0.002	0.003	<0.001	<0.001	<0.001
Constipation	CMT (N = 61)	28.4 (35.9)	32.8 (34.7)	35.5 (35.9)	37.0 (32.7)	35.2 (35.2)	32.1 (36.4)
	IMT (N = 50)	5.3 (18.3)	8 (20.8)	9.3 (23.4)	8.9 (26.0)	4.9 (15.9)	3.6 (15.3)
	*p*	<0.001	<0.001	<0.001	<0.001	<0.001	<0.001
Diarrhea	CMT (N = 61)	3.8 (15.0)	5.5 (17.4)	2.7 (9.2)	9.6 (19.6)	5.7 (18.9)	4.9 (15.2)
	IMT (N = 50)	4.7 (19.0)	5.3 (17.0)	6.7 (17.8)	10.4 (17.2)	16.3 (26.0)	18.9 (26.7)
	*p*	0.938	0.986	0.291	0.574	0.021	0.016
Financial difficulties	CMT (N = 61)	33.9 (30.7)	36.6 (28.4)	38.8 (32.9)	34.8 (26.6)	32.4 (29.7)	25.9 (25.0)
	IMT (N = 50)	19.3 (26.2)	14.0 (19.2)	16.0 (22.6)	12.6 (19.2)	12.2 (19.4)	13.5 (20.0)
	*p*	0.009	<0.001	<0.001	<0.001	0.001	0.037

SD = standard deviation; CMT = chemotherapy; IMT = immunotherapy.

## Data Availability

The data presented in this study are available on reasonable request from the corresponding author.
